# Effect of Different Thawing Regimes on Cell Kinematics and Organelle Integrity of Nitrogen-Stored Wallachian Ram Spermatozoa

**DOI:** 10.3390/vetsci11120602

**Published:** 2024-11-27

**Authors:** Martin Ptáček, Filipp Georgijevič Savvulidi, Christopher LeBrun, Martina Janošíková, Temirkhan Kenzhebaev, Kairly Omashev, Beybit Kulataev, Nurlan Malmakov

**Affiliations:** 1 Department of Animal Science, Faculty of Agrobiology, Food and Natural Resources, Czech University of Life Sciences Prague, Suchdol, 165 00 Prague, Czech Republic; savvulidi@af.czu.cz (F.G.S.); xlebc001@studenti.czu.cz (C.L.); janosikova@af.czu.cz (M.J.); 2 Meat Sheep Breeding Department, Kazakh Research Institute of Livestock and Fodder Production, Zhandosov Str., Bldg. 51, Almaty 050 035, Kazakhstan; kterdesh@mail.ru (T.K.); okairly@mail.ru (K.O.); bnar68@mail.ru (B.K.); nurlan_malmakov@mail.ru (N.M.)

**Keywords:** cryopreservation, insemination doses, ovine, CASA, flow cytometry, spermatozoa intactness

## Abstract

It is well known that the suboptimal thawing of frozen insemination doses critically reduces the fertilizing ability of spermatozoa and, consequently, the effectiveness of artificial insemination. In this study, we aimed to determine the optimal thawing procedure for frozen Wallachian ram insemination doses. The study used a standard method of obtaining ejaculates from rams, diluting semen with a commercial extender, and a common methodology for freezing sperm in straws with minor modifications. After storage in liquid nitrogen, doses were thawed using several regimes: straws were thawed at 45, 50, 55, 60, and 65 °C for durations of 2, 5, 8, 11, and 14 s for each temperature. The thawing regime at 39 degrees for 30 s was chosen as a reference thawing method, to which all others were compared. Using a computer-assisted sperm analyser and flow cytometry, the motility, kinematic parameters, and spermatozoa cell organelle damage of the thawed samples were analysed and supplemented with an analysis of the internal temperature gradient and thermal energy absorption of each straw. Based on the obtained results, we concluded that the thawing regime at 39 degrees for 30 s is the preferred regime for ram sperm. Our study may be useful with regard to standardizing the procedure for the thawing of frozen ram sperm insemination doses and, therefore, might significantly improve the effectiveness of artificial insemination techniques in the sheep industry.

## Simple Summary

The thawing procedure represents an essential, but still ununified, step in the cryoconservation process. For this reason, the aim of our study was to conduct a detailed evaluation of the effect of 25 different thawing protocols (different temperatures and thawing durations) on the kinematic parameters and damage to sperm cell organelles compared to the reference thawing method (39 °C/30 s). Insemination doses from Wallachian ram spermatozoa were uniformly prepared (same rams, semen collection technique, semen handling, preparation, and freezing of semen doses). Thawed semen samples were analysed using a computer-assisted sperm analyser (kinematic analyses) and flow cytometry (cell organelle integrity). A concurrent supplemental analysis of each straw’s internal temperature gradient and thermal energy absorption was also performed. Our results indicate that the thawing regime at 39 °C for 30 s was the preferred regime for ram sperm. Our study may be a useful tool for standardizing and simplifying the thawing procedure of frozen ram sperm insemination doses.

## 1. Introduction

Artificial insemination is an advanced reproductive technology used to increase the number of lambs born from elite sires to accelerate genetic gain in a flock [1]. Insemination doses stored in liquid nitrogen also represent an essential tool for creating a gene reservoir of threatened livestock species, which contributes to biodiversity [2,3]. However, to this day, the process of cryopreservation damages sperm cells dramatically, as sperm cells are exposed to two critical thermal zones: first, when frozen to −196 °C, and second, when thawed [4,5,6,7]. The thawing process is as critical for sperm survivability as the initial equilibration and freezing procedure of sperm cryopreservation [8]. All aspects of the cryopreservation process are of great interest to researchers, with the goal of optimizing the procedure and improving the success of sperm cell preservation. As a result, the detailed monitoring of cryopreserved sperm thawing appears to be a crucial step in the whole methodological procedure. The importance and perspectives of thawing procedures for different livestock species were highlighted in a recently published review by Pezo et al. [9].

However, the procedure for sheep sperm thawing has not yet been standardized. Paulenz et al. [10] thawed insemination doses frozen in 0.25 mL straws at 70 °C for 7 to 8 s. Another Scandinavian study published by Söderquist et al. [11] investigated different thawing regimes of ram sperm (70 °C/5 s vs. 50 °C/9 s vs. 35 °C/12 s). Their results indicated that 70 °C/5 s and 50 °C/9 s thawing protocols reached similar results. In contrast, post-thaw sperm motility with regard to the percentage of cells with an intact membrane was significantly higher for straws thawed at 70 °C/5 s (67.0% motility and 50.5% membrane intactness) than those thawed at 35 °C/12 s (63% motility and 41.7% membrane intactness), as reported by Söderquist et al. [11]. Rapid thawing protocols were routinely applied or suggested in previous studies: 65 °C for 6 s [12,13], 70 °C for 5 s [14], or 70 °C/8 s [15]. Conversely, Evans and Maxwell [16] recommended thawing ram spermatozoa at 38–42 °C for 15–30 s, which resulted in comparable sperm motility, acrosome integrity, and sperm cell fertilization capacity. This method of thawing is considered a reference method that is commonly used (directly or with minor modifications) in cryopreservation procedures, as documented in a number of studies: 37 °C/30 s [17]; 37 °C/12 s [18]; 35 °C/12 s [19]; 37 °C/30 s [20]; 37 °C/30 s [21]; and 37 °C/25 s [22]. Thawing at 37–38 °C also simulates the female body temperature and is the most widely used, not only in in vivo conditions but in in vitro studies as well, as Pezo et al. [9] have explored.

Thawing temperatures and duration are the two determining criteria in thawing protocols. These variables are manifested by varying thawing intensities, which are characterized by changes in the temperature gradient within the straw and the total thermal energy absorbed by the insemination dose during the thawing process. Especially at higher thawing temperatures, a prolonged thawing time results in massive sperm deterioration [23]. Daghigh et al. [24] warned that a temperature of 60 °C, even for a duration as short as 4 s, caused significant cell damage and reduced cell motility. As thawing protocols are a very complex interaction of temperatures and time intervals, over which substantial damage can occur very quickly, they have not yet been analysed in enough detail that the correct thawing strategy can be clearly set/verified. Additionally, the broader implications regarding changes in the temperature gradient within doses and the total amount of thermal energy absorbed have not yet been studied within the context of thawing protocols, which can provide a more complete and complex assessment of the viability of post-thaw sperm cells when combined with the kinematic parameters and cell organelle integrity results.

The main aim of this study was to perform a detailed evaluation (including an analysis of sperm kinematic parameters and cell organelle damage) of the effect of different thawing protocols (doses were tested across a wide range of temperatures and various thawing time intervals, with each temperature being tested with every time interval uniformly) on the motility and organelle integrity of Wallachian ram spermatozoa compared to the reference thawing method (39 °C/30 s), making this the most expansive analysis performed on thawing temperatures and durations thus far. An additional aim of this study was to quantify the internal temperature gradient of the straws while thawing under each of the defined protocols, as well as to estimate each straw’s total energy absorption.

## 2. Materials and Methods

### 2.1. Reagents and Solutions

Dulbecco’s phosphate-buffered saline (PBS; D8537) (–Ca/–Mg) was obtained from Sigma-Aldrich (Merck KGaA, Darmstadt, Germany). ANDROMED^®^ (Minitüb GmbH, Tiefenbach, Germany) served as the freezing extender. Hoechst-33342 (H-342; 14533) and propidium iodide (PI; P4170) were purchased from Sigma-Aldrich (St. Louis, MO, USA). Alexa Fluor 488-labelled lectin PNA from Arachis hypogaea (PNA; L21409) and MitoTracker™ Deep Red FM (MTR-DR; M22426) were sourced from Thermo Fisher Scientific (Waltham, MA, USA).

### 2.2. Rams and Sperm Collection

Ejaculates were collected multiple times from three mature Wallachian rams with typical exterior signs of the breed and excellent breeding history. All rams were transported from an area of the Beskids Mountains and kept under identical breeding conditions at the Demonstration and Experimental Centre of the Czech University of Life Sciences in Prague (GPS coordinates: 50°07′47.6″ N 14°22′07.0″ E). Semen collection and evaluation were conducted with a minimal time delay to reduce variability. All rams were maintained under identical conditions with strict veterinary monitoring. The animals had access to both barn shelters and fenced grazing areas. Their diet consisted of unrestricted access to pasture and hay, supplemented with commercial concentrate pellets (1580 OVCE UNI, De Heus a.s., Bučovice, Czech Republic; BMK TEX O, VVS Verměřovice s.r.o., Czech Republic) at 0.25–1.00 kg per animal daily, adjusted based on the body condition score. Mineral licks (Solsel, Mikrop Čebín a.s., Czech Republic) and water were provided ad libitum. The health status and body condition score (BCS 3.5–4.0) were monitored throughout the study [25].

Semen was naturally collected using a sheep/goat artificial vagina (AV) (Minitübe GmbH, Tiefenbach, Germany) prepared according to the manufacturer’s instruction manual. Semen was collected at the end of a natural mating season (November–February period). After collection, the ejaculate was visually assessed (volume, colour, odour, foreign impurities: urine and blood elements). If the samples met the criteria, they were placed in a thermobox with a temperature between 25 and 30 °C and transported to the laboratory for further analysis and processing (within 30 min after semen collection, transportation using this method maintains semen quality and adheres to the protocol established by Jurado-Campos et al. [26], which was subsequently validated and optimized under our laboratory conditions).

### 2.3. Sperm Samples Analyses

Mass motility was determined in the laboratory for each sample [27]. Only samples with a mass motility of three or higher were evaluated for sperm concentration using a spectrophotometer (Genesys™ 10vis, Thermo Fisher Scientific, Waltham, MA, USA). After the concentration assessment, the sperm was diluted with the AndroMed^®^ extender (Minitüb GmbH, Tiefenbach, Germany) to a final concentration of 200 million sperm/mL. The ejaculate was filled into 0.25 mL straws and sealed with polyvinyl alcohol (IMV Technologies, L’Aigle, France). Equilibration was carried out in a refrigerator at 5–10 °C for 2–4 h.

### 2.4. Sperm Freezing

The straws were frozen in liquid nitrogen vapour using a freezing box [28] and a freezing curve previously optimized for freezing Wallachian ram spermatozoa [29]. The frozen straws were kept in the liquid nitrogen for several days before thawing.

### 2.5. Semen Thawing

**Figure 1:**
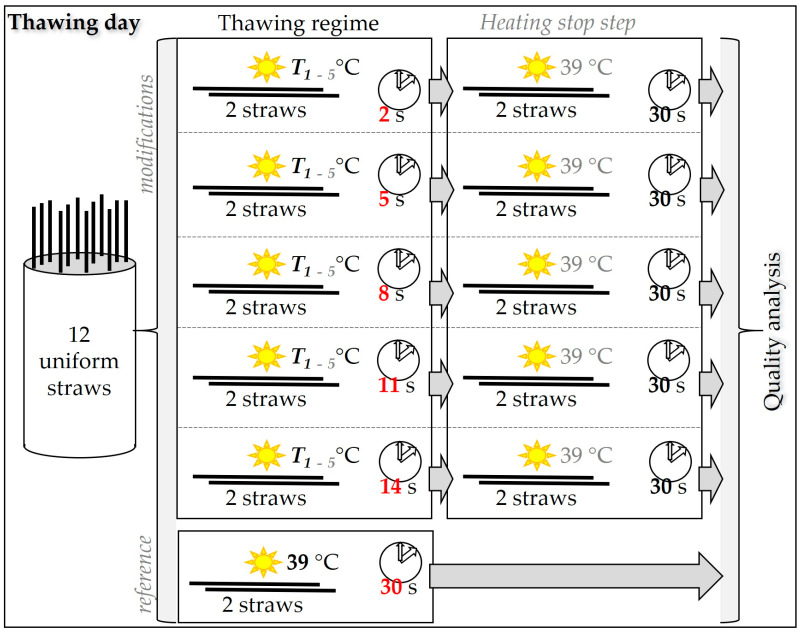
Scheme of semen thawing procedure design. T_1–5_ = thawing temperature (where T_1_—45 °C; T_2_—50 °C; T_3_—55 °C; T_4_—60 °C; T_5_—65 °C).

The straws, uniformly frozen according to the above methodical description, were thawed in a digital unstirred water bath (VWR International s.r.o., Stříbrná Skalice, Czech Republic) according to thawing protocols characterized by different temperatures and durations of thawing. In order to verify the modified thawing protocols, the straws were thawed at 45, 50, 55, 60, and 65 °C for durations of 2, 5, 8, 11, and 14 s for each temperature. After thawing, the straws were immediately transferred to water at a temperature of 39 °C, where it was kept for 30 s to stop the heating of the contents of the straw. A thawing procedure of 39 °C for 30 s was used as a reference thawing protocol for all the defined variants [16]. This procedure was performed on each experimental thawing day. A total of 12 straws were thawed per experimental thawing day, with always two straws per variant (thus, 5 modifications and 1 reference variant were tested per day). Specific thawing protocols were tested repeatedly, at least 4× on different experimental thawing days, so that the data set was sufficiently coherent to be objectively statistically evaluated. The sequence of particular methodical steps was illustrated in Figure 1 for a better visualization of the thawing procedure design.

After thawing, sperm quality characteristics, sperm motility, kinematic parameters, and spermatozoa cell organelle damage were analysed at two intervals. The first measurement was immediately after thawing (T0), and the second measurement was performed 2 h after incubation in a water bath tempered at 39 °C (T2).

### 2.6. Sperm Kinematic Analysis

Sperm kinematic parameters were analysed using iSperm^®^ mCASA (Aidmics Biotechnology, Taipei, Taiwan) following the manufacturer’s protocols. Briefly, the semen was diluted with the extender to a final concentration of 30–60 million cells/mL; the 7.5 µL drop was placed on a chip and analysed using Ovine 5 application. A minimum of three fields per iSperm sample chip were evaluated. The following sperm kinematic parameters were assessed: total motility (TM, %), progressive motility (PM, %), average path velocity (VAP, μm/s), curvilinear velocity (VCL, μm/s), straight-line velocity (VSL, μm/s), linearity (LIN, %), and straightness (STR, %).

### 2.7. Flow Cytometric Assay

Samples were diluted in PBS to 20 × 10^6^ spermatozoa/mL, transferred to a 96-well plate, and incubated with freshly prepared fluorescent dyes for 10 min at 38 °C in darkness. The final dye concentrations were as follows: H-342 (10 μg/mL) to assess DNA content, PI (8 μg/mL) to assess plasma membrane damage, PNA (0.5 μg/mL) to assess acrosome damage, and MTR DR (80 nM) to assess mitochondrial activity [30]. Subsequently, sperm samples were analysed using a NovoCyte digital flow cytometer, model 3000 (Acea Biosciences, part of Agilent, Santa Clara, CA, USA). The flow cytometer was equipped with three solid-state lasers: violet (405 nm, 50 mW) for H-342, blue (488 nm, 60 mW) for PI and PNA, and red (640 nm, 40 mW) for MTR-DR excitation [31]. Calibration beads (NovoCyte, QC Particles, Agilent Technologies, Santa Clara, CA, USA) were used to calibrate the flow cytometer before analysing the samples. Flow cytometric analysis was performed at low speed, evaluating a minimum of 10,000 sperm cells per sample. NovoExpress software, v1.3.0 (Acea Biosciences, Agilent, Santa Clara, CA, USA) was used for automated cytometer setup, performance tracking, and data acquisition. The data were saved and subsequently analysed by the same software. No compensation was required for the optical filter settings used. The flow cytometric gating strategy and population hierarchy were adopted without modifications from Savvulidi et al. [29]. The resulting cluster of events was initially identified using a side scatter (SSC) versus forward scatter (FSC) bivariate histogram plot. Spermatic events were identified based on the gating set with the H-342 stain. Spermatic events with an intact plasma membrane and acrosome were identified based on the PI and PNA signal intensities. Finally, viable sperm were evaluated by the combination of the above-mentioned fluorescent markers with MTR-DR positive cells (H-342+/PNA-/PI-/MTR DR+).

### 2.8. Internal Thermal Gradient and Thermal Energy Absorption of Straws During Thawing

An HH506A digital thermometer, equipped with a “K type” thermocouple (Omega Engineering Inc., Norwalk, CT, USA), was used to record temperature changes directly inside the straw during each (modified or reference) thawing procedure.

A thermocouple was placed in each dose during the freezing process, and then temperature changes were recorded every 1 s from the frozen state until the dose was fully thawed.

The total thermal energy absorbed during the thawing process was calculated by analysing the temperature changes in the insemination doses. The heat transfer equation (Q = mc*ΔT) was used to determine the energy (in Joules) absorbed by a single straw under different thawing regimes. For this calculation, the heat capacity of ice was approximated to be 1500 kJ/kgK.

### 2.9. Statistical Analysis

A statistical evaluation was performed using SAS software (SAS/STAT^®^ 9.3, 2011). Generalised linear models (GLMs) were used to assess statistical differences between the thawing procedures and sperm kinematic (TM, PM, VAP, VCL, VSL, LIN, STR) and cell damage (VIA, PAD, PMD, ACD, MIT) parameters at times T0 and T2. All these relationships were corrected simultaneously to determine the effect of the semen collection day.

In this sense, two-factorial ANOVA with fixed effects of the day of semen collection (1st semen collection day, *n* = 18; 2nd semen collection day, *n* = 36; 3rd semen collection day, *n* = 18; 4th semen collection day, *n* = 54; 5th semen collection day, *n* = 54; 6th semen collection day, *n* = 36; 7th semen collection day, *n* = 18; 8th semen collection day *n* = 18; 9th semen collection day, *n* = 18; 10th semen collection day, *n* = 36; 11th semen collection day, *n* = 18; 12th semen collection day, *n* = 36; and 13th semen collection day, *n* = 36) and thawing procedure (39 °C/30 s, *n* = 66; 45 °C/2 s, *n* = 15; 45 °C/5 s, *n* = 15; 45 °C/8 s, *n* = 15; 45 °C/11 s, *n* = 15; 45 °C/14 s, *n* = 15; 50 °C/2 s, *n* = 15; 50 °C/5 s, *n* = 15; 50 °C/8 s, *n* = 15; 50 °C/11 s, *n* = 15; 50 °C/14 s, *n* = 15; 55 °C/2 s, *n* = 12; 55 °C/5 s, *n* = 12; 55 °C/8 s, *n* = 12; 55 °C/11 s, *n* = 12; 55 °C/14 s, *n* = 12; 60 °C/2 s, *n* = 12; 60 °C/5 s, *n* = 12; 60 °C/8 s, *n* = 12; 60 °C/11 s, *n* = 12; 60 °C/14 s, *n* = 12; 65 °C/2 s, *n* = 12; 65 °C/5 s, *n* = 12; 65 °C/8 s, *n* = 12; 65 °C/11 s, *n* = 12; 65 °C/14 s, *n* = 12) was performed.

Statistically significant differences were defined according to the Tukey–Kramer test at a significance level of *p* < 0.05.

## 3. Results

The aim of this study was to identify kinematic parameters and the intensity of damage to sperm cell organelles when applying different thawing strategies. The modified protocols tested were always compared to a reference thawing procedure: 39 °C/30 s.

### 3.1. Sperm Kinematic Analysis

**Figure 2:**
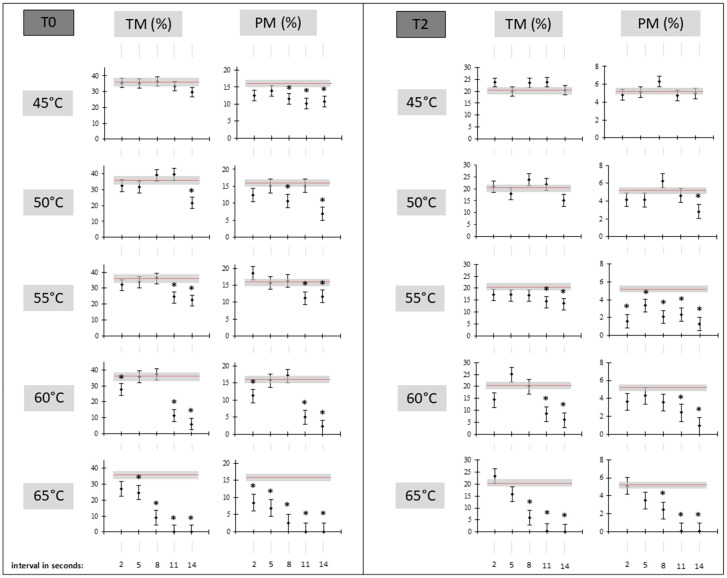
Total (TM) and progressive (PM) motility modified thawing protocols compared to the reference (39 °C/s) one. T0 = results detected immediately after thawing; T2 = results detected after 2 h of incubation in a water bath heated at 39 °C; black points with bounded lines indicate LSM values ± SE for modified thawing protocols; the red line with a grey marked area indicates the reference thawing protocol (LSM ± SE); * = indicates a significant difference in the modified thawing protocol to the reference thawing protocol at *p* < 0.05 level of significance.

No indication of improvement in total or progressive motility was detected for the tested thawing modifications versus the reference thawing procedure, as seen in Figure 2. Progressive and total motility immediately after thawing and after 2 h of incubation reached similar (with no significant evidence) or even significantly lower results compared to the reference thawing procedure. Generally, significantly lower results were more frequently detected for protocols with a higher temperature (55 °C and higher) or with a prolonged time of thawing (8 s and longer), as shown in Figure 2. Additionally, no other evaluated kinematic parameter showed a clear positive trend to support the said protocol as a routine thawing strategy (Figures S1 and S2). The typical shape for these traits was demonstrated by the identification of some potential positive tendencies (non-significant or with statistically significant support) followed by a rapid drop. This was very clearly visible in the evaluated traits immediately after thawing. However, the results at T2 were also markedly lower than the reference thawing procedure in a predominant number of cases, and rapid drops to nearly 0 values were visible as well (especially for the thawing procedure with a 65 °C temperature and a duration of 11 or more seconds).

### 3.2. Flow Cytometry Assay

**Figure 3:**
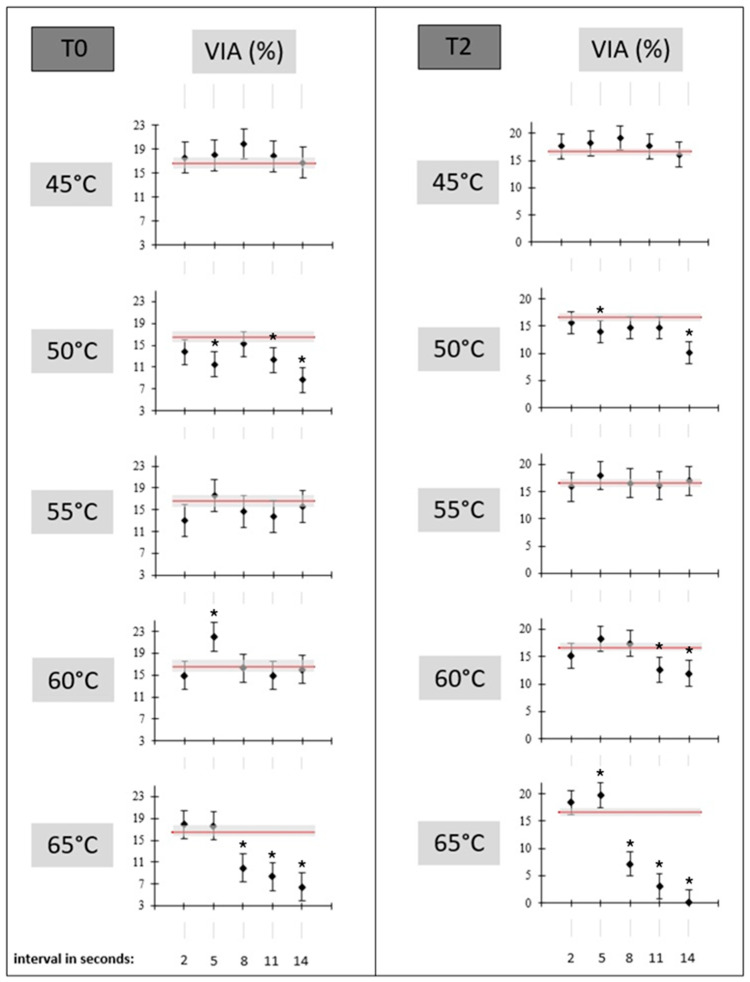
Sperm viability (VIA) of modified thawing protocols compared to the reference (39 °C/s) one. T0 = results detected immediately after thawing; T2 = results detected after 2 h of incubation in a water bath heated at 39 °C; black points with bounded line indicate LSM values ± SE for modified thawing protocols; the red line with a grey marked area indicates the reference thawing protocol (LSM ± SE); * = indicates a significant difference in the modified thawing protocol to reference the thawing protocol at *p* < 0.05 level of significance.

According to our flow cytometry analysis results, no superiority of alternative thawing regimes compared to the reference (39 °C for 30 s) based on sperm viability was detected. This was clear immediately after thawing and after two hours of incubation at 39 °C as well (Figure 3). The only exceptions were 60 °C for 5 s, which reached 5.42% higher sperm viability at the time of thawing, and 65 °C for 5 s after 2 h of incubation with a 3.10% increase in viable sperm with regard to the reference thawing protocol. Moreover, the output results of the alternative thawing procedures compared to the reference had no clear onset trend or progression trend that could be interpreted as positive, and thus, the alternative thawing regimes might not be recommended as a thawing procedure. On the contrary, the thawing modifications more frequently resulted in lower sperm viability and more damage to the cells’ structure. Spermatozoa were prone to plasma membrane damage and lower mitochondrial activity when temperatures increased to 60 °C (Figures S3 and S4). This represents a major risk when considering any thawing regime with high temperatures. Additionally, no modified variation in the thawing regimes achieved higher mitochondrial activity in comparison to the reference thawing procedure. In other words, all thawing regime alternatives reached similar or significantly lower mitochondrial activity, which is also clear in Figures S3 and S4.

### 3.3. Internal Thermal Gradient and Thermal Energy Absorption of Insemination Doses

**Figure 4:**
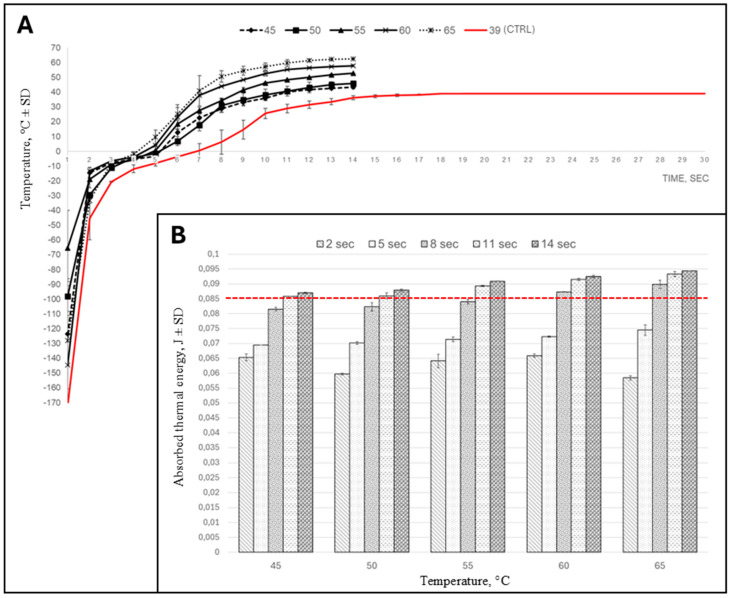
(**A**)—Graphical illustration of the thawing curves for different thawing regimes. The temperature change inside the straw during thawing is shown. The red line represents the thawing curve of the control regime (39 degrees for 30 s). (**B**)—The approximation of thermal energy (calculated in Joules) absorbed by a single straw at different thawing regimes. The red dotted line represents the energy absorbed by a single straw at a reference thawing regime (39 degrees for 30 s).

Figure 4A shows the internal straw temperature throughout the thawing process. In general, massive temperature increases were observed during the first 2 s of thawing, followed by a somewhat moderate increase between 2 and 5 s, and another more noticeable increase from 5 to 10 s. After 10 s of thawing, the temperature inside the straws stabilized. As expected, the results showed that thawing temperatures had a direct effect on the internal temperature of the straws and, as such, the reference thawing protocol (39 °C/30 s) exhibited the lowest heat change in comparison to the modified thawing procedures.

In contrast, the total amount of absorbed heat was determined by the duration of thawing rather than the temperature. This phenomenon is clearly evident in Figure 4B, which shows that time intervals up to 8 s, regardless of thawing temperature, achieved a lower or comparable amount of heat absorption compared to the reference thawing protocol (39 °C/30 s). Overall, the highest total absorbed thermal energy was evident at temperatures of 55, 60, and 65 °C for thaw durations of 11 and 14 s.

## 4. Discussion

Wallachian sheep represent an endemic population of traditional sheep breeds, which are strongly protected under a government rescue programme [32]. This illustrates the importance of this type of research for various livestock and wild species. Additionally, it is in alignment not only with national conservation programmes but also international programmes, including the One Conservation concept [33], where cryopreservation processes underscore the significance of combining ex situ and in situ conservation efforts to help maintain genetic variability and support the restoration of species in their natural environments [33]. The thawing procedure is a crucial step in the whole cryoconservation process, and for this reason, different thawing strategies have been largely investigated for pigs and cattle livestock species. The systematic monitoring of different temperatures at different time intervals was performed by Tomas-Almenar and Mercado [34] with boar semen and, more recently, by Alvarez-Rodriguez et al. [35]. Both studies agree independently that rapid thawing procedures (70 °C/8 s) are the best for thawing Iberian boar semen. Eriksson and Rodriguez-Martinez [36] found significantly higher motility with rapid thawing protocols (70 °C/8 s or 50 °C/13 s) in comparison to 35 °C/23 s frozen semen stored in FlatPacks packages. However, no simultaneously significant differences in plasma membrane intactness across different thawing procedures were detected [36]. Nur et al. [37] observed sperm motility and sperm defects under different thawing treatments in Holstein bulls. A rapid thawing procedure (70 °C/5 s) resulted in significantly higher motility and significantly lower acrosomal and morphological defects in comparison to 50 °C/15 s and 37 °C/30 s thawing protocols. The positive effect of rapid thawing protocols was confirmed with the sperm kinematic analysis of Holstein and Czech Fleckvieh bull spermatozoa by Doležalová et al. [38]. Unfortunately, studies on the thawing procedure of frozen ram sperm are scarce. Actually, only a few studies involved in thawing protocols for frozen ram spermatozoa exist. A positive effect of rapid thawing procedures was detected by Söderquist et al. [11]. Their results on ram sperm showed 63% motility and 50% membrane intactness after thawing at 35 °C for 12 s [11]. Furthermore, the rapid thawing procedure (70 °C/5 s) resulted in a 4% (*p* < 0.05) higher motility and an 8.8% (*p* < 0.05) higher membrane intactness in comparison with 35 °C/12 s. In spite of some indication for improvement in both kinematic parameters and sperm organelle damage with alternative thawing strategies, our results cannot be generalized as with all the previous studies. We were unable to make a definitive recommendation for alternative thawing regimes, as the results were generally lower and showed no clear trend when comparing the modified thawing protocols to the reference protocol. Conversely, quite a clear negative tendency was observed during prolonged durations of thawing (more than 11 s), especially in temperatures exceeding 55 °C. This significantly increased the risk of severe cell damage and indicated the potential risk factor posed when considering rapid thawing rates. Our results are not sporadic in their interpretation, and Daghigh et al. [24] reached similar conclusions to those of ours. Their results did not find significant differences when testing 37 °C/30 s vs. 60 °C/6 s thawing protocols for sperm movement characteristics, viability, plasma membrane integrity, and total sperm abnormality. Additionally, no differences were detected as far as lipid peroxidation, glutathione peroxidase, superoxide dismutase, and the total antioxidant capacity with the two thawing rates are concerned. For the purpose of optimizing thawing protocols for Wallachian sheep, we can agree with the previously established recommendations published by Evans and Maxwell [16], who, as a standard, recommended thawing ram spermatozoa at a temperature of 38–42 °C for 15–30 s. This thawing procedure is safe with better or at least comparable results to all systematic modifications. Additionally, this procedure is supported by the approaches of Paulenz et al. [39] and Nordstoga et al. [40], who evaluated fertility outcomes (25-day non-return or lambing rates) following insemination with doses thawed using different protocols. Neither study observed the significant effects of thawing procedures on fertility results after artificial insemination, and as a result, Nordstoga et al. [40] concluded that the non-significant difference in ewes’ fertility gives rise to the possibility of introducing a simplified and more rational one-step protocol (37 °C/15 or 20 s) for ram semen processing. Furthermore, these results rather indicate that the conclusive improvements detected in vitro may not work in the same way in vivo conditions do.

Doležalová et al. [38] have also pointed out the importance of monitoring the persistence of sperm when comparing different thawing strategies. Although their results showed the positive effect of rapid thawing protocols on kinematic parameters immediately after thawing, the outcomes of their persistence test (2 h of incubation in a water bath temperature at 39 °C) were comparable across all thawing protocols with the higher temperatures that were tested. Moreover, the best numerical results were observed for the standard 39 °C/30 s protocol. Notably, our findings remained consistent both immediately after thawing and after 2 h of incubation post-thaw. These results not only align with those of Doležalová et al. [38], but they also provide empirical support for the 39 °C/30 s protocol, highlighting its benefits not only for sperm resistance but also for persistence, offering a broader perspective on thawing protocols.

In order to better understand what occurred within the insemination doses, we supplemented our study with data about the internal temperature gradients and overall energy absorption of each insemination dose. Although thermal energy absorption progressively increased with thawing durations from 2 to 14 s (providing results both lower and higher than the reference method), the temperature gradient for all modified thawing protocols was higher than that of the reference method. While keeping in mind the results of the kinematic analysis and of cell organelle damage, it seems evident that the temperature gradient, which was more subtle for the reference thawing method, appears more significant than the amount of thermal energy absorbed.

Regarding our methodological approach and interpretation of results, it should be mentioned that our choice of AndroMed^®^ as a diluent was based on its demonstrated cryoprotective efficacy in previously published studies on ram and goat ejaculate cryopreservation. The choice of extender used can significantly affect the outcome of the ram semen freezing process, and there are currently a number of commercially available extenders for freezing ram semen. The extender used in our study was AndroMed^®^ (Minitube, Germany), which is egg-yolk-free. This extender has been used to freeze ram semen in a significant number of previously published studies and has proven to be successful at improving ram semen cryopreservation results [41,42,43,44].

However, we understand that there clearly cannot be a one-fits-all extender that is suitable for all ram breeds, and as such, the extender we chose (AndroMed^®^) cannot be claimed as the best extender for ram sperm in general. This can be confirmed by the results of a study where, after comparing several extenders used during the freezing of ram sperm from two different breeds, AndroMed^®^ did not show the best results [45]. Nonetheless, we are aware that different diluents provide different protective effects [46], and as such, we cannot exclude the fact that thawing protocols with alternative diluents may theoretically interact differently.

The results of our study provide a comprehensive comparison of an important process for insemination dose manipulation. This innovative approach establishes stronger connections between the physical properties of heat and energy transfer during the thawing process, and these results are valuable for a complete understanding of the thawing process, as well as for future studies involving thawing protocols.

## 5. Conclusions

Rams are among the most susceptible livestock species to spermatozoa damage during the cryopreservation process. As a result, thawing protocols represent an important tool in the complex methodological approach for insemination dose handling. Despite a very detailed comparison of various thawing protocols (supported by the precise measurements of temperature gradients and thermal energy absorption within insemination doses during the thawing process) against the 39 °C/30 s reference method, no clear interpretable significant improvement in sperm kinematic characteristics or organelle damage was observed from using these modified thawing strategies. Some indications of potential improvement were identified. A more in-depth examination of other sheep breeds might have revealed more significant and stable differences, providing clearer evidence of improvement. In any case, we can not confirm this improvement based on our results. Therefore, for the cryopreservation of ram ejaculate from Wallachian sheep, the reference thawing protocol of 39 °C for 30 s is recommended as a simple, safe, and sufficiently effective method for handling insemination doses.

## Data Availability

Data are available within this article.

## Supplementary Materials

The following supporting information can be downloaded at https://www.mdpi.com/article/10.3390/vetsci11120602/s1. Figure S1: Additional kinematic parameters of modified thawing protocols compared to the reference (39 °C/s) one detected immediately after thawing; Figure S2: Additional kinematic parameters of modified thawing protocols compared to the reference (39 °C/s) one detected after 2 h of incubation; Figure S3: Sperm plasma membrane damage, acrosomal damage, and mitochondrial activity of modified thawing protocols compared to the reference (39 °C/s) one detected immediately after thawing; and Figure S4: Sperm plasma membrane damage, acrosomal damage, and mitochondrial activity of modified thawing protocols compared to the reference (39 °C/s) one detected after 2 h of incubation.
