# Supplementary figures and images for: Effect of Different Thawing Regimes on Cell Kinematics and Organelle Integrity of Nitrogen-Stored Wallachian Ram Spermatozoa
Source: Vet Sci. 2024 Nov 27;11(12):602. doi: 10.3390/vetsci11120602 (PMC11680395; doi:10.3390/vetsci11120602)

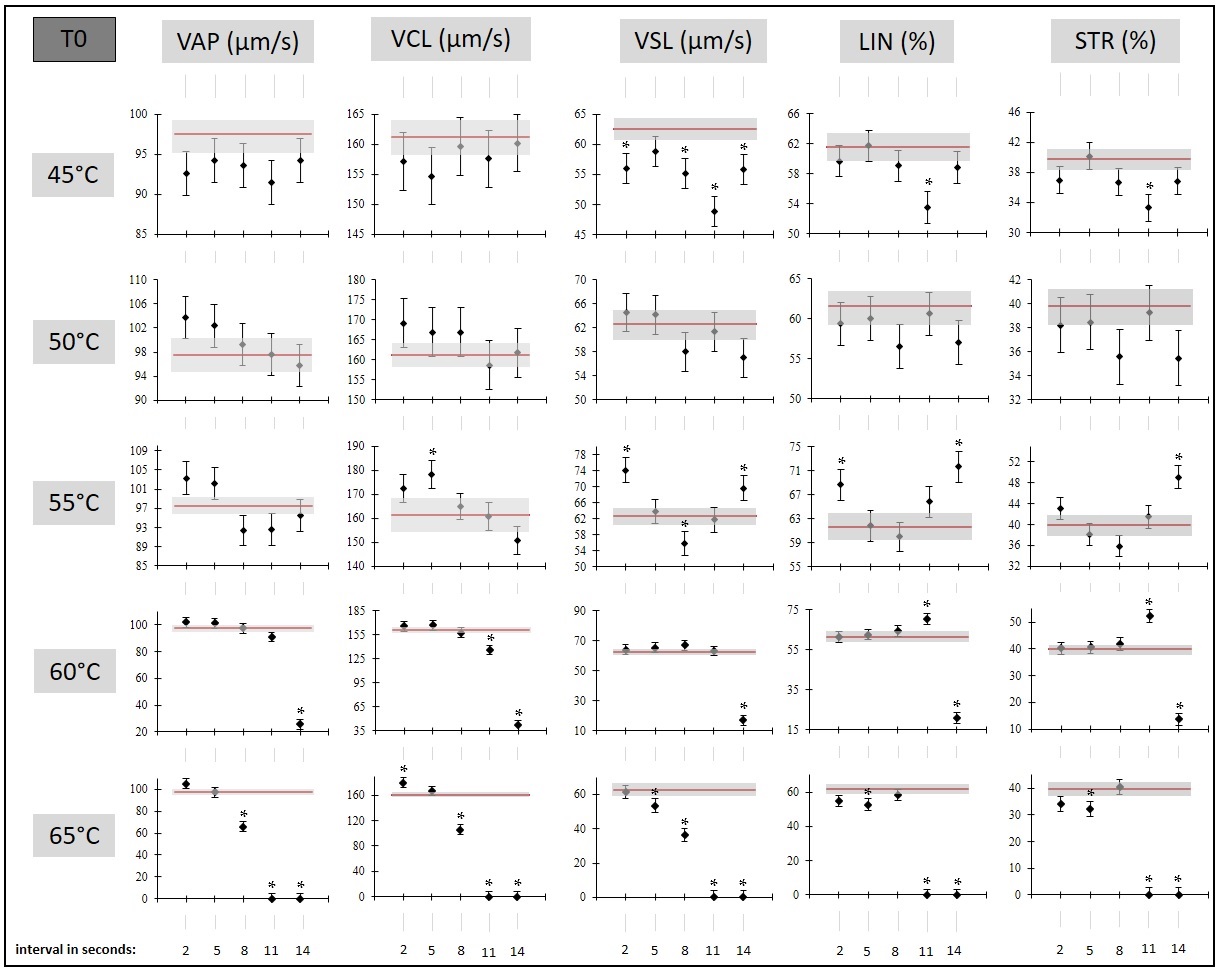

Supplement: Supplementary file 1 [file vetsci-11-00602-s001.zip › Figure S1.jpg]

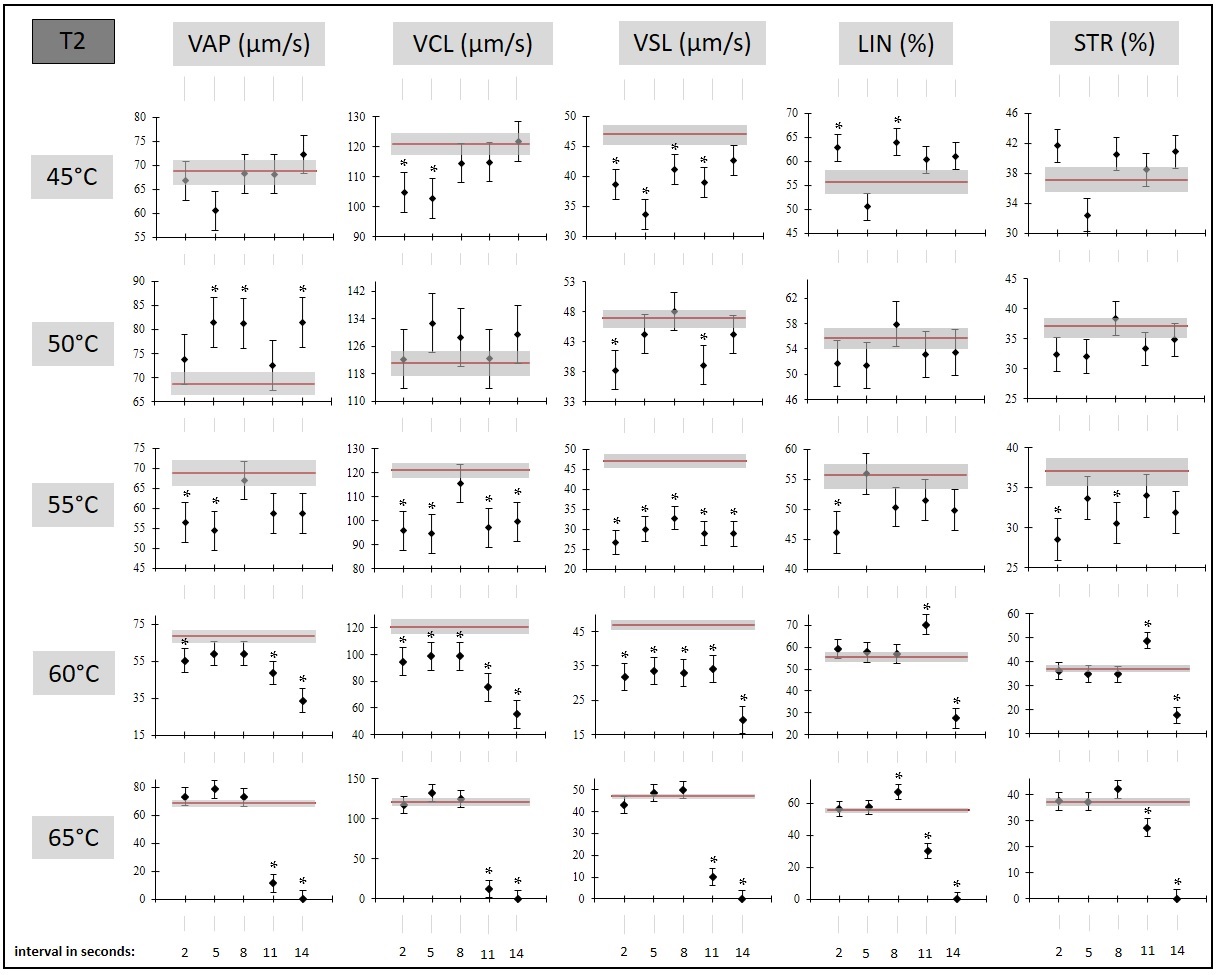

Supplement: Supplementary file 1 [file vetsci-11-00602-s001.zip › Figure S2.jpg]

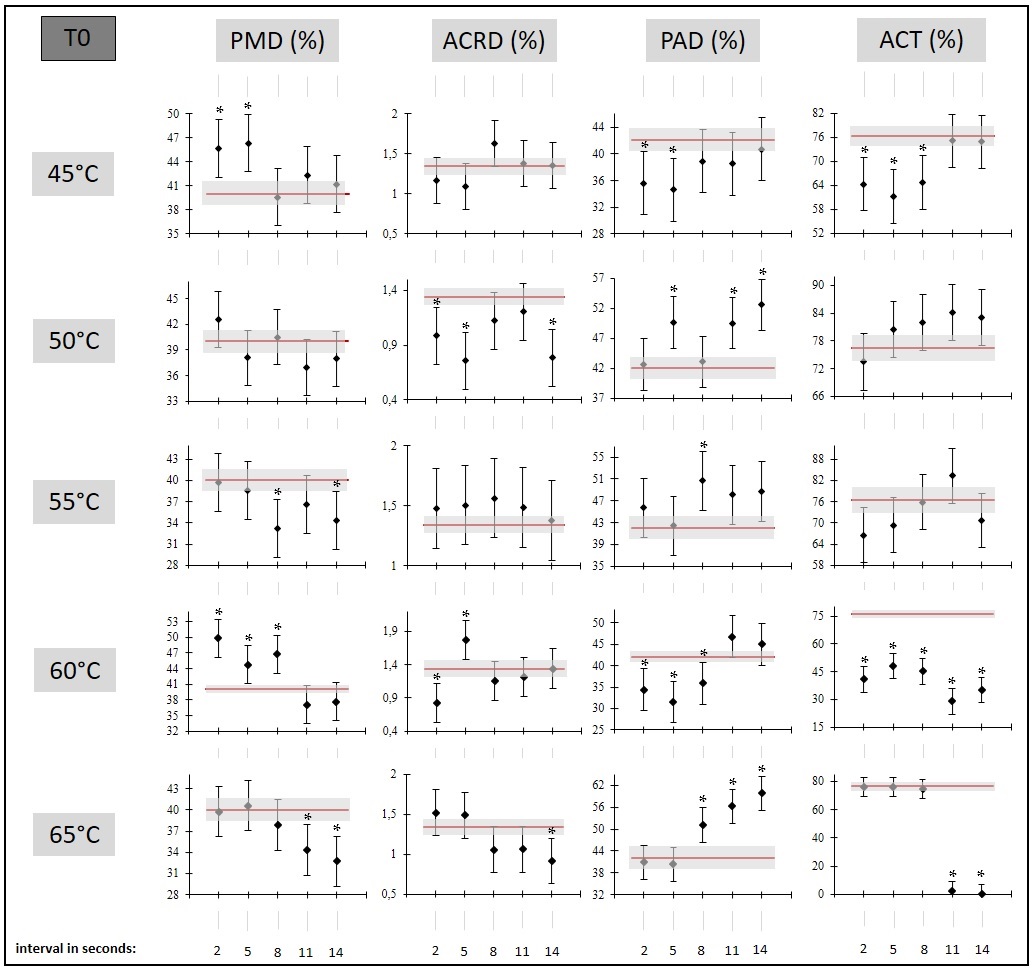

Supplement: Supplementary file 1 [file vetsci-11-00602-s001.zip › Figure S3.jpg]

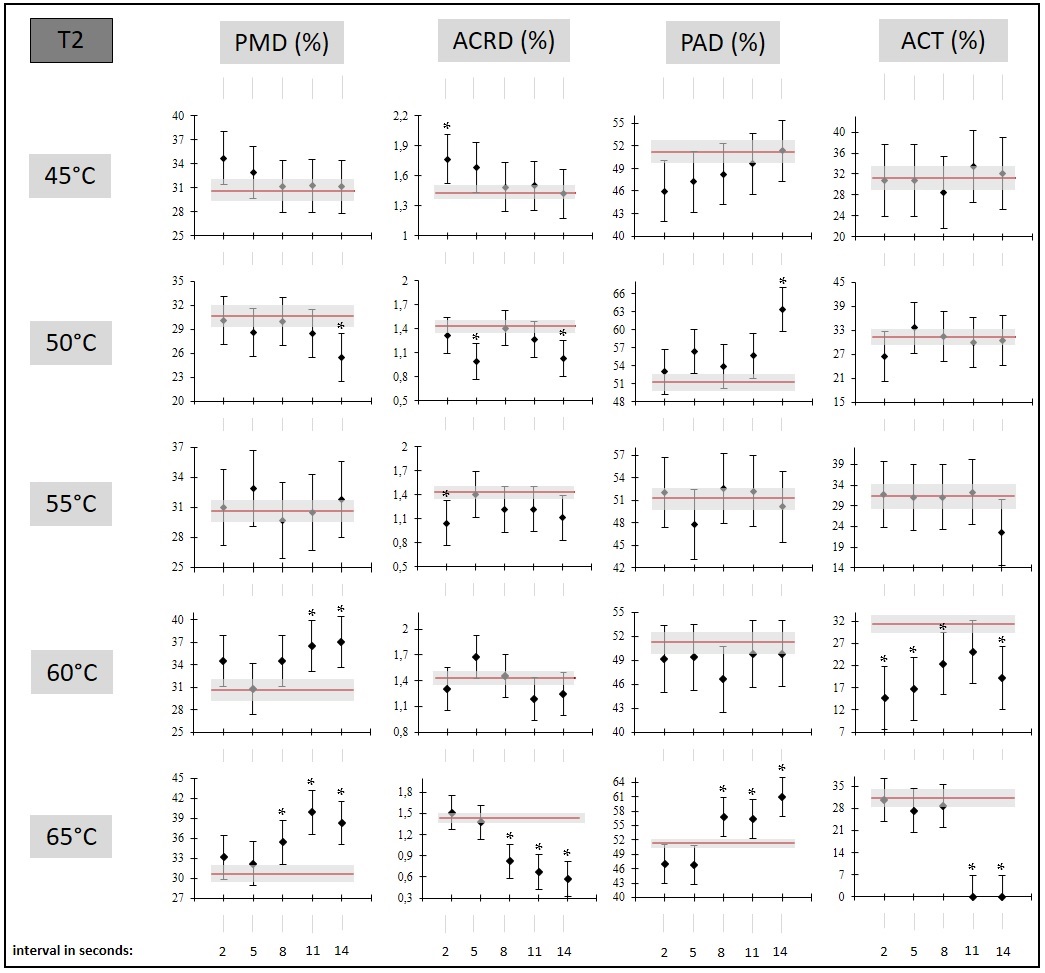

Supplement: Supplementary file 1 [file vetsci-11-00602-s001.zip › Figure S4.jpg]
